# Experience Affects EEG Event-Related Synchronization in Dancers and Non-dancers While Listening to Preferred Music

**DOI:** 10.3389/fpsyg.2021.611355

**Published:** 2021-04-12

**Authors:** Hiroko Nakano, Mari-Anne M. Rosario, Constanza de Dios

**Affiliations:** ^1^Department of Psychology, Saint Mary's College of California, Moraga, CA, United States; ^2^Department of Physics and Astronomy, Saint Mary's College of California, Moraga, CA, United States; ^3^Center for Neurobehavioral Research on Addiction, Department of Psychiatry and Behavioral Sciences, University of Texas Health Science Center, Houston, TX, United States

**Keywords:** music, expert, dance, EEG, alpha, beta, gamma, time frequency analysis (TFA)

## Abstract

EEGs were analyzed to investigate the effect of experiences in listening to preferred music in dancers and non-dancers. Participants passively listened to instrumental music of their preferred genre for 2 min (Argentine tango for dancers, classical, or jazz for non-dancers), alternate genres, and silence. Both groups showed increased activity for their preferred music compared to non-preferred music in the gamma, beta, and alpha frequency bands. The results suggest all participants' conscious recognition of and affective responses to their familiar music (gamma), appreciation of the tempo embedded in their preferred music and emotional arousal (beta), and enhanced attention mechanism for cognitive operations such as memory retrieval (alpha). The observed alpha activity is considered in the framework of the alpha functional inhibition hypothesis, in that years of experience listening to their favorite type of music may have honed the cerebral responses to achieve efficient cortical processes. Analyses of the electroencephalogram (EEG) activity over 100s-long music pieces revealed a difference between dancers and non-dancers in the magnitude of an initial alpha event-related desynchronization (ERD) and the later development of an alpha event-related synchronization (ERS) for their preferred music. Dancers exhibited augmented alpha ERD, as well as augmented and uninterrupted alpha ERS over the remaining 80s. This augmentation in dancers is hypothesized to be derived from creative cognition or motor imagery operations developed through their dance experiences.

## Introduction

Experts are people who are trained in a field, such as a sport, art, or any type of physical or mental task. The difference between experts and novices can be most readily observed in their performance outcomes. Differences between these groups may also be seen in electrophysiology. For example, in a seminal work, Buzsáki (2006) describes the effects of long-term meditation practice on electroencephalogram (EEG), namely an increase in the power of the alpha oscillation that extends from the occipital to central regions. Meditation is an intense mental practice to enhance attentional skills, wherein experts employ the technique of “tuning into your inner-self but still remaining aware of the surroundings” (Buzsáki, 2006). The alpha oscillation correlates with the degree of training, suggesting that experts have an increased level and expanded spatial distribution of alpha activity.

Human alpha oscillation was first observed in EEG of a conscious person in a relaxed state of mind (Berger, 1929 cited in Jasper and Carmichael, 1935). Based on the observations of alpha's appearance over the occipital area (i.e., vision) when eyes were closed and the disappearance when eyes were open, alpha is historically regarded as “cortical idling” (e.g., Adrian and Matthews, 1934; Chase and Harper, 1971), implying a default brain mode when cortical neurons are not functioning (Pfurtscheller et al., 1996). However, if increased alpha activity indicates “cortical idling,” it is surprising that alpha is observed in meditation, a practice of attention which requires neuronal activation. In this sense, it is “paradoxical” that alpha activity shows up both in a relaxed state of mind and during intense internal focus (Buzsáki, 2006).

Alpha activity in experts may also be viewed within the framework of the neural efficiency hypothesis (e.g., Grabner et al., 2003; Del Percio et al., 2009; Babiloni et al., 2010). For example, Del Percio et al. (2009) found that alpha activity was more resistant to reduction in elite karate and fencing athletes than non-athletes when they performed simple monopodalic and bipodalic standing tasks. The authors propose that the high level of alpha activity is indicative of less cortical neural activity, implying that experts can produce task performances at a level equal to non-experts even with a higher level of cortical idling. This indicates that some neural motor commands underlying body movement and coordination required less cortical activation for this physical task in expert athletes compared to non-athletes.

Similar results were observed during non-motor cognitive tasks in athletes. Compared to non-athletes, higher alpha activity was observed in expert athletes while judging the level of karate skills of athletes on video (Babiloni et al., 2010); in acrobatic gymnasts, while viewing gymnastic videos with or without erroneous movements (Ivaldi et al., 2018); and in karate practitioners, while performing arithmetic subtraction (Duru and Assem, 2018). These results from motor and cognitive tasks may suggest general neural efficiency in expert athletes.

Alpha increase has also been observed in expert musicians. Professional and amateur musicians listened to the same musical piece and were tasked to rate the moment-to-moment emotional arousal level of the music, while their EEG were recorded. Increased alpha activity was found in the professional musicians during the subjective high-arousing segment of the music, suggesting enhanced arousal processing in music among these experts (Mikutta et al., 2014).

In this study, we examined expert Argentine tango dancers to investigate the effect of dance experience on their electrophysiological (EEG) response to tango music and explored the corresponding cognitive processes. To focus on the dance expertise effect, dancers listened to their preferred tango music and non-dancers their own preferred music, jazz, or classical, making each group the expert of their own music. There was no specific task in this experiment. As dance experts, dancers may voluntarily engage in mental activities unique to dancers. We expected that the mental activity would be reflected in an increase of alpha activity, which would suggest focused attention or enhanced emotional arousal as other types of experts have shown. We expected that the alpha effect in dancers would be more robust compared to non-dancers while both groups engage in listening to their own preferred music as experts.

The expected expertise effect would not be observed only in the increase of alpha activity but also in the temporal development of alpha activity. We explored the question of how and when expertise-related processes emerge and develop over an extended period of time (100 s). Although previous studies have investigated expert dancers' EEG responses to dance-related tasks, no studies have looked at changes in patterns over a long period of time. For example, though expert dancers showed a change in EEG, e.g., an alpha decrease when the dancers evaluated dance movements shown in a video, the time period of the EEG measurement was limited to 6 s (Orgs et al., 2008). The 6-s window of investigation may not be long enough because in music listening, it has been reported that a self-recognition of emotional arousal takes more than 8 s in both expert musicians and non-musicians; and that the time course differs depending on the familiarity and types of music (Bachorik et al., 2009). Considering affective influences on music perception, we anticipated that spontaneous EEG responses would change over relatively long time.

## Methods

### Participants, Stimuli, and EEG Recording

Sixteen participants (range: 20–48 years old, *M* = 37.8 years, *SD* = 6.78 years; eight female, eight male) were dancers who regularly practiced Argentine Tango (range 5–17 years, *M* = 9.2 years, *SD* = 3.5 years), and had varying degrees of musical training (range 0-20 years, *M* = 5.3 years, *SD* = 5.9 years). Sixteen participants (range 20–61 years, *M* = 32.6 years, *SD* = 16.1 years; eight female., eight male) were enthusiasts of jazz (*N* = 8) or classical music (*N* = 8) who regularly listened to their genre (range 5–46 years, *M* = 18 years, *SD* = 13 years), had no dance training, and had varying degrees of musical training (range 0–47 years, *M* = 10.9 years, *SD* = 14.5 years). All participants were right-handed, with no neurological conditions. All provided written informed consent. The Institutional Review Board of Saint Mary's College of California approved this study.

Each participant provided three pieces of favorite instrumental music. Tango dancers (dancer group) provided tango pieces and non-dancers (non-dancer group) jazz or classical pieces. Each piece was cut to 122 s long, fading out over the last 2 s. Nine total distinct pieces—three tango, three jazz/classical, and three instrumental foxtrot as filler pieces—were presented in three blocks. Each block contained a tango, jazz/classical, and filler in random order. Dancers listened to their own music (tango), randomly selected filler pieces, and jazz/classical music randomly selected from a collection of jazz/classical pieces provided by the researchers and those brought in by non-dancers. Half the dancers received jazz and the other half classical music. Non-dancers listened to their own music (jazz/classical), randomly selected filler pieces, and tango music randomly selected from a collection of tango pieces provided by the researchers and those brought in by dancers. Half the non-dancers brought jazz and the other half classical music.

Participants listened to the music through over-ear headphones (Beyerdynamic DTX900) with eyes closed while sitting in a dimly-lit sound-attenuated room. They were asked not to move their eyes and body. Participants' hearing ability was not formally assessed, and volume setting was not standardized across participants. Instead, participants were asked to choose a comfortable level of volume at the beginning of the session and during the session. Participants sat at rest, listening to silence, for two minutes at the start of each block. This “rest” condition served as the baseline for subsequent EEG analysis. To implicitly elicit neuronal responses, no specific tasks were given; participants were told to enjoy the music. Participants rated their enjoyment of each piece right after it was presented on a scale of 1–10 (10 = most enjoyable). After the EEG session, participants were interviewed about their listening experience.

EEG was recorded from 32 sintered Ag/AgCl electrodes mounted in an elastic cap (Neuroscan 32 QuickCap). Electrodes placed below and above the left eye, and in the right and left canthi, monitored eye movement. The derivation of electrodes was based on the common reference method. Electrodes were referenced to linked right and left mastoids. Impedances were kept <5 kΩ. Data was acquired at 1,000 Hz, and online bandpass filtered from 0.05 to 100 Hz (Neuroscan SynAmp I).

### Data Analysis

EEG data were downsampled to 320 Hz and high-pass filtered at 0.20 Hz. Trials were 120.100s long (0.100s pre-stimulus). Non-stereotyped artifacts were visually identified and rejected. An average of 1.11s was removed per trial; trials with > 12.0s of rejected data were excluded from analysis; as a result, the first 100s of data of each trial was further analyzed. Independent component analysis was performed on each participant's data. Components related to muscle activity and eye movement were identified and removed (Jung et al., 2000).

To determine the alpha, beta, and gamma bands, we performed a spectral analysis of each participant's EEG data using the Welch method, with 500 ms windows, a Hanning taper, and 50% overlap; windows were zero padded to obtain spectral estimates every 0.125 Hz. A histogram of the frequency peak positions for all participants in all conditions showed that peaks were grouped into the ranges 8.000–12.625 Hz (alpha), 15.125–22.625 Hz (beta), and 26.250–56.000 Hz (gamma).

We further calculated the event-related synchronization (ERS) for each participant. In our study, the “event” is defined as the cognitive event of listening to the musical stimulus. ERS compares the power of the EEG in an experimental condition relative to the power of EEG during a reference condition, in our case the “rest” condition. In this type of spectral analysis, power reflects the degree of neural oscillatory synchrony within a particular scalp region (Pfurtscheller, 2003; Womelsdorf and Fries, 2007). Positive ERS values, resulting from relative increases in power, are considered synchronization. Negative ERS values, resulting from relative decreases, are considered desynchronization (ERD).

ERS was determined from a time-frequency analysis (TFA) of each participant's data, performed within EEGlab (Delorme and Makeig, 2004), which computes the event related spectral perturbation (ERSP) at regular time points, in our case every 250 ms. ERSP used the same parameters given above, and did not include a pre-stimulus baseline subtraction or division. The power in each band was calculated by averaging the ERSP over the frequencies within the ranges given above. ERSP in the music conditions were compared to the rest condition by subtracting the ERSP during the rest at each time point, *ERSP*(*t*_i_)_music_−*ERSP*(*t*_*i*_)_rest_, and this difference was normalized using the time averaged rest ERSP for each participant:

$$\begin{array}{l} ERS\left(t\right)=\;\frac{{ERSP\left(t_{i}\right)}_{music}-{ERSP\left(t_{i}\right)}_{rest}}{\frac{1}{N}\sum_{i=1}^{N}{ERSP\left(t_{i}\right)}_{rest}}100\% \end{array}$$

where *i* indexes the time point, and *N* is the total number of time points. The resulting values are considered the event-related synchronization at different time points, *ERS*(*t*), for each participant.

*ERS*(*t*) values were divided and averaged into five 20 s-long intervals (0−20 s, 20−40 s, …) to visualize the results and to investigate the temporal development. Preliminary analyses using different interval lengths (20, 10, 5, and 2.5 s) showed that the *ERS*, averaged over all electrode sites^1^, had qualitatively similar time development across choices of time bins. Furthermore, topographic plots of 10.0 and 20.0 s interval lengths were qualitatively similar as well. The 20 s interval was chosen to make the number of comparisons in the subsequent analyses of variance (ANOVA) more manageable and comprehensible.

*ERS* values, averaged over trials and over time, were subjected to mixed analyses of variance (ANOVA) with Group (dancer, non-dancer) as a between-subjects factor and Music (tango, non-tango), Interval (1: 0−20 s, 2: 20−40 s, 3: 40−60 s, 4: 60−80 s, 5: 80−100 s) and Electrodes (30 sites) as within-subjects factors. Filler pieces were not included in the analysis. The mixed ANOVAs were performed separately for each frequency band. Within-subjects factors were applied with Greenhouse-Geisser correction. The Greenhouse-Geisser-corrected *F* values are reported with uncorrected degrees of freedom. Significant interactions with Music were followed up with Bonferroni-corrected *t*-tests.

Musicianship was not considered as a factor, as years of musical training showed no significant difference between the two groups (*p* = 0.17) and were not correlated with *ERS* values in any of the frequency bands (all *p*-values > 0.50). Age and gender were also not considered as factors due to the lack of difference between groups [Age: *t*(20.2) = 1.21, *p* = 0.24; Gender: χ^2^(1) = 0, *p* = 1.00] and lack of correlation with *ERS* values in any of the frequency bands (Age: all *p*-values > 0.11; Gender: all *p*-values > 0.08).

## Results

**Figure 1:**
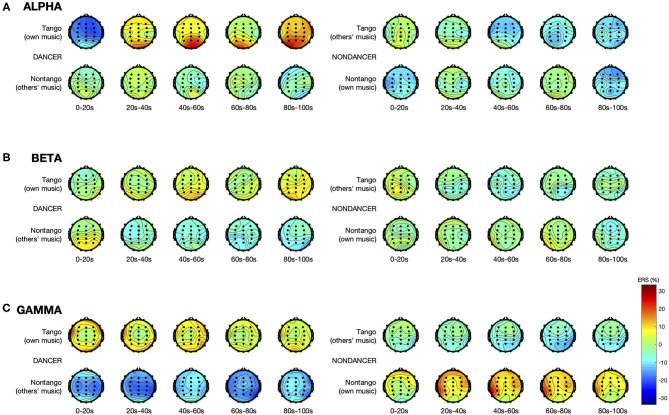
*ERS* in the **(A)** alpha, **(B)** beta, and **(C)** gamma bands. Each topographic plot represents mean *ERS* calculated over a 20-s long interval, averaged over all trials within each music condition, and all participants within each group. Electrode sites, represented by the dots, are at standard positions given by the international 10–20 system.

**Figure 2:**
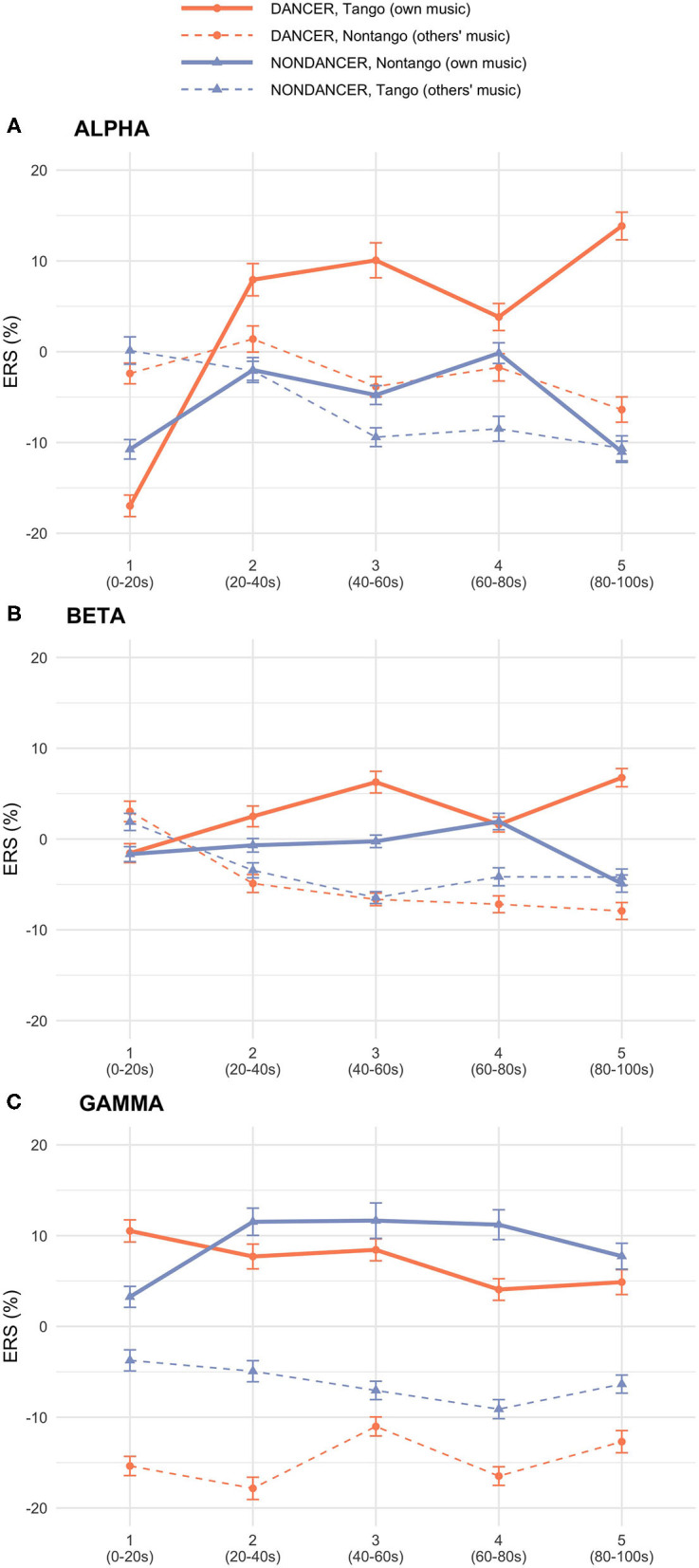
Group × Music × Interval interaction plots of mean *ERS* in **(A)** alpha, **(B)** beta, **(C)**, and gamma bands. Line plots show mean *ERS* over each time Interval within each Music condition, per Group.

Figure 1 illustrates the spatial distribution and time course of *ERS* in the alpha, beta, and gamma bands. Figure 2 displays the electrode-averaged *ERS* at each interval in the alpha, beta, and gamma bands.

### Alpha

There was a significant Group × Music × Interval interaction [*F*_(4,120)_ = 8.18, *p* < 0.001, $\eta_{p}^{2}$ = 0.21] and a main effect of Electrode [*F*_(29,870)_ = 4.04, *p* = 0.002, $\eta_{p}^{2}$ = 0.12]. See Figures 1A, 2A. The absence of a main effect of Music [*F*_(1,30)_ = 0.70, *p* = 0.41] suggests that differences in intrinsic music features such as tempo, meter, and rhythm were unlikely to have played a role in either group's alpha activity.

*Post-hoc* comparisons are provided in Table 1, using Bonferroni-corrected *t*-tests. Table 1A shows comparisons between Groups as they listened to their own music (tango in dancers vs. non-tango in non-dancers) at each interval, using independent-samples *t*-tests. Table 1B shows comparisons between Music conditions within each Group (for example, dancers listening to tango vs. dancers listening to non-tango) at each interval, using paired-samples *t*-tests.

**Table 1A:** Group-wise comparison: *Post-hoc* tests of significant Group × Music × Interval interactions in each band.

		**Alpha**				**Beta**				**Gamma**
	**Time interval**	***t***	***p***	***d***	**Group with higher activity**	***t***	***p***	***d***	**Group with higher activity**	
Own music (tango in dancers minus non-tango in non-dancers)	1	**−3.89**	** <0.001**	**−0.25**	**Non-dancer**	0.09	0.932	0.01	Dancer	No significant Group × Music × Interval interaction
	2	**4.43**	** <0.001**	**0.29**	**Dancer**	**2.33**	**0.020**	**0.15**	**Dancer**	
	3	**6.78**	** <0.001**	**0.44**	**Dancer**	**4.74**	** <0.001**	**0.31**	**Dancer**	
	4	**2.13**	**0.034**	**0.14**	**Dancer**	**–**0.27	0.787	**–**0.02	Non-dancer	
	5	**12.95**	** <0.001**	**0.84**	**Dancer**	**8.47**	** <0.001**	**0.55**	**Dancer**	
Other's music (non-tango in dancers minus tango in non-dancers)	1	**–**1.33	0.182	**–**0.09	Non-dancer	0.79	0.432	0.05	Dancer	
	2	**1.97**	**0.0496**	**0.13**	**Dancer**	**–**1.13	0.260	**–**0.07	Non-dancer	
	3	**3.67**	** <0.001**	**0.24**	**Dancer**	**–**0.21	0.836	**–**0.01	Non-dancer	
	4	**3.32**	**0.001**	**0.21**	**Dancer**	**−2.23**	**0.026**	**−0.14**	**Non-dancer**	
	5	**2.18**	**0.030**	**0.14**	**Dancer**	**−2.90**	**0.004**	**−0.19**	**Non-dancer**	

*Bonferroni-corrected independent-sample t-tests compared each group's own music to each other (tango in dancers minus non-tango in non-dancers) at each Interval, shown in the upper rows. Comparisons between each group when listening to the other groups' music (non-tango in dancers minus tango in non-dancers) are in the bottom rows. Significant comparisons (p-values <0.05) are in boldface. Cohen's d indicates effect size that can be interpreted qualitatively as follows (Cohen, 1988): 0.2 small, 0.5 medium, 0.8 large*.

**Table 1B:** Music-wise comparison: *Post-hoc* tests of significant Group × Music × Interval interactions in each band.

		**Dancer**				**Non-dancer**			
**Band**	**Time interval**	***t***	***p***	***d***	**Music condition with higher activity**	***t***	***p***	***d***	**Music condition with higher activity**
Alpha	1	**−14.07**	** <0.001**	**−0.64**	**Non-tango**	**13.12**	** <0.001**	**0.60**	**Tango**
	2	**4.41**	** <0.001**	**0.20**	**Tango**	**–**0.09	0.928	**–**0.004	Non-tango
	3	**7.86**	** <0.001**	**0.36**	**Tango**	**−4.34**	** <0.001**	**−0.20**	**Non-tango**
	4	**3.78**	** <0.001**	**0.17**	**Tango**	**−7.27**	** <0.001**	**−0.33**	**Non-tango**
	5	**11.56**	** <0.001**	**0.53**	**Tango**	0.43	0.671	0.02	Tango
Beta	1	**−5.89**	** <0.001**	**−0.27**	**Non-tango**	**5.77**	** <0.001**	**0.26**	**Tango**
	2	**8.35**	** <0.001**	**0.38**	**Tango**	**−4.39**	** <0.001**	**−0.20**	**Non-tango**
	3	**14.20**	** <0.001**	**0.65**	**Tango**	**−8.76**	** <0.001**	**−0.40**	**Non-tango**
	4	**10.94**	** <0.001**	**0.50**	**Tango**	**−7.99**	** <0.001**	**−0.36**	**Non-tango**
	5	**14.04**	** <0.001**	**0.64**	**Tango**	1.08	0.280	0.05	Tango
Gamma	No significant interaction for Group × Music × Interval								

*Bonferroni-corrected paired-samples t-tests (t) compared levels of Music within each Interval per Group. That is, tango minus non-tango in dancers, and tango minus non-tango in non-dancers. Positive t-values indicate higher activity in the tango condition relative to non-tango, while negative t-values indicate the reverse. Significant comparisons (p-values <0.05) are in boldface. Cohen's d indicates effect size that can be interpreted qualitatively as follows (Cohen, 1988): 0.2 small, 0.5 medium, 0.8 large*.

*Post-hoc* analysis of the main effect of electrode identified that the posterior electrodes O1, Oz, and O2 were most active when compared with each of the other 29 electrodes (*p*-values < 0.001, 0.011, and 0.046, respectively).

### Beta

There were significant interactions of Group × Music [*F*_(1,30)_ = 6.53, *p* = 0.016, $\eta_{p}^{2}$ = 0.18], Group × Music × Interval [*F*_(4,120)_ = 6.66, *p* = 0.001, $\eta_{p}^{2}$ = 0.18], and Group × Music × Electrode [*F*_(29,870)_ = 3.83, *p* = 0.003, $\eta_{p}^{2}$ = 0.11]. See Figures 1B, 2B. The lack of a main effect of Music [*F*_(1,30)_ = 2.11, *p* = 0.16] suggests that differences in intrinsic music features such as tempo, meter, and rhythm were unlikely to have played a role in either group's beta activity.

*Post-hoc* tests for the Group × Music interaction *via* Bonferroni-corrected paired-samples *t*-tests indicated higher *ERS* in the tango condition compared to non-tango for the dancer group [*t*(2,399) = 18.62, *p* < 0.001, *d* = 0.38], and higher *ERS* in the non-tango condition compared to tango for the non-dancer group [*t*(2,399) = −6.89, *p* < 0.001, *d* = −0.14].

*Post-hoc* comparisons using Bonferroni-corrected *t*-tests for the Group × Music × Interval interaction for beta are shown in Table 1. Table 1A shows comparisons between groups as they listened to their own music (tango in dancers vs. non-tango in non-dancers) at each interval, using independent-samples *t*-tests. Table 1B shows comparisons between music conditions within each group (for example, dancers listening to tango vs. dancers listening to non-tango) at each Interval, using paired-samples *t*-tests.

### Gamma

There were significant interactions of Group × Music [*F*_(1,30)_ = 27.73, *p* < 0.001, $\eta_{p}^{2}$ = 0.48], and Group × Music × Electrode [*F*_(29,870)_ = 3.00, *p* < 0.001, $\eta_{p}^{2}$ = 0.09]. See Figures 1C, 2C. The lack of a main effect of Music [*F*_(1,30)_ = 0.85, *p* = 0.37] suggests that differences in intrinsic music features such as tempo, meter, and rhythm were unlikely to have played a role in either group's gamma activity.

*Post-hoc* comparisons for the Group × Music interaction indicated higher *ERS* in the tango condition relative to non-tango in dancers [*t*(2,399) = 37.81, *p* < 0.001, *d* = 0.77], and higher *ERS* in the non-tango condition relative to tango in non-dancers [*t*(2,399) = −24.94, *p* < 0.001, *d* = −0.51].

Comparisons between each Group's own Music (tango in dancers vs. non-tango in non-dancers) *via* Bonferroni-corrected independent-samples *t*-tests showed higher gamma activity in non-dancers when they listened to non-tango, compared to dancers when they listened to tango [*t*(4615.5) = −2.17, *p* = 0.03, *d* = −0.06]. Non-dancers also showed higher gamma activity when they listened to tango, compared to dancers when they listened to non-tango [*t*(4790.7) = −12.10, *p* < 0.001, *d* = −0.35].

### Behavioral

**Figure 3:**
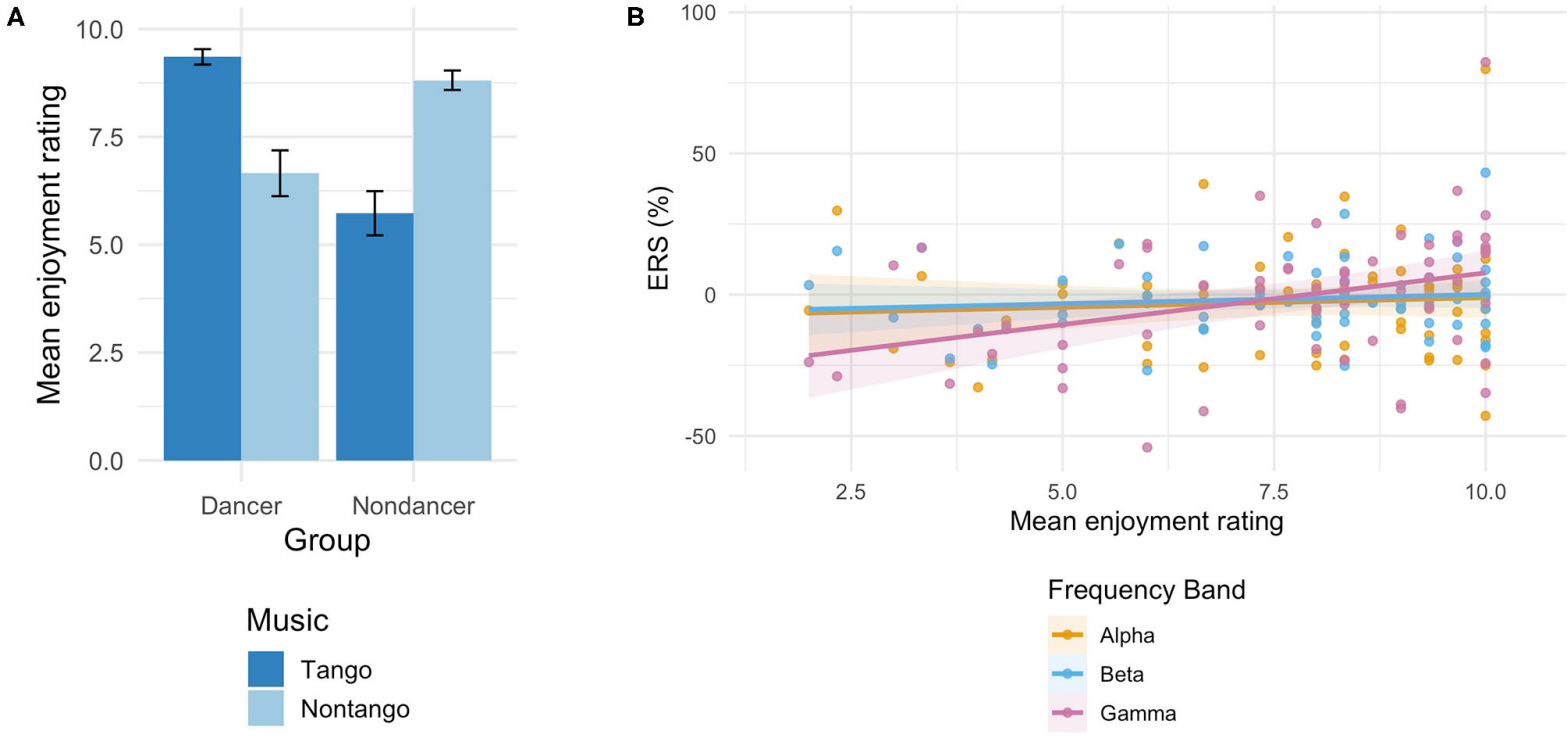
**(A)** Mean enjoyment ratings of music (1 = no enjoyment, 10 = most enjoyable) by music condition in each participant group. Error bars represent standard error of the mean. Tango condition served as dancers' own music, while non-tango condition served as non-dancers' own music. **(B)** Estimated rating scores for all tango and non-tango music as a linear function of *ERS* in all participants for the alpha, beta, and gamma bands. Shaded regions indicate confidence intervals at each estimate. A linear mixed regression excluding extreme values (±3*SD* from mean *ERS*) did not alter the findings [Main effect of ERS *p*-values in original analysis: *p* = 0.991 (alpha), *p* = 0.695 (beta), *p* = 0.011 (gamma); Main effect of ERS *p*-values in analysis without outliers: *p* = 0.388 (alpha), *p* = 0.888 (beta), *p* = 0.012 (gamma)].

Both groups rated their own music higher than the other group's music on the enjoyment scale (1 = no enjoyment, 10 = most enjoyable). See Figure 3A. Mean rating scores for their own music were 9.4 ± 0.2 (dancers) and 8.8 ± 0.2 (non-dancers); and for the other group's music 6.7 ± 0.5 (dancers) and 5.7 ± 0.5 (non-dancers). A Group × Music ANOVA on the ratings yielded a significant Group × Music interaction [*F*_(1,30)_ = 61.4, *p* < 0.001, $\eta_{p}^{2}$ = 0.67], signifying higher ratings for their own music in both groups.

To test the relation of the spectral activity with the enjoyment ratings of the music, a linear mixed regression was performed on rating scores for all tango and non-tango music using *ERS* values, and Group (dancer, non-dancer), as fixed-effects factors, and participant as a random-effects factor. Overall model fit was assessed in each frequency band by comparing the null regression (intercept-only) model against the full model (with factors ERS and Group) with an *F*-test with Satterthwaite approximation for degree of freedom (Luke, 2017). Figure 3B shows marginal estimates produced by the overall models of enjoyment scores as a function of *ERS* in each frequency band. Results showed that ratings were significantly predicted by *ERS* values in both groups only in the gamma band [Main effect of ERS: *t*(60.0) = 2.63, *p* = 0.011], and the full model was significantly different from the null model in the gamma band [χ^2^(3) = 12.30, *p* = 0.006]. *ERS* values did not predict ratings in the alpha or beta bands (*p* > 0.623); the full model was not significantly different from the null model in these bands [alpha: χ^2^(3) = 2.32, *p* = 0.509; beta: χ^2^(3) = 2.72, *p* = 0.437].

## Discussion

Our analysis revealed an expertise effect across the three bands in both dancers and non-dancers. The Group × Music interaction in the gamma and beta bands showed higher activity for tango music in dancers, and for non-tango music in non-dancers. That is, both groups' gamma and beta activities were strongest when listening to their own music. Multiple regression analyses revealed an association between gamma activity and music enjoyment judgements. The Group × Music × Interval interaction in the alpha band similarly indicated the expertise effect exhibiting both groups' significant changes in time-dependent alpha activity during listening to their own music: alpha ERD in the first interval followed by ERS in the later time intervals. However, the change in alpha activity over time was more robust in dancers compared to non-dancers. The initial alpha decrease and later alpha increase were stronger and more sustained in dancers than in non-dancers. In the following section, we will discuss how our participants' expertise in preferred-music listening was reflected in gamma, beta, and alpha bands. The robustness of alpha activity in dancers could reflect an augmented expertise effect that has emerged as a result of dance experiences.

### Expertise Effect in Higher Frequencies

Higher frequencies, namely gamma and beta, are generally associated with wakefulness, perceptual alertness and conscious cognitive operations (e.g., Buzsáki, 2006). An increase of activity in gamma oscillations is particularly associated with tasks that involve selective attention, working memory (WM), and conscious stimulus recognition (Müller and Keil, 2004; Pavlova et al., 2006; Womelsdorf and Fries, 2007; Carozzo et al., 2010). For example, selective attention studies showed that magnetoencephalogram gamma power elicited by attended color was larger than that elicited by non-attended color in color checkerboard sequences (Müller and Keil, 2004); and similarly, for attended motion (walking human figures) compared to unattended motion (Pavlova et al., 2006). In a somatosensory WM study (Haegens et al., 2010) where participants were told to remember the frequency of electric pulses delivered to their hand in order to match a probe pattern after a delay, gamma during the retention period was stronger for correct trials compared to incorrect. Other WM studies showed a similar pattern whereby the degree of gamma activity predicted behavioral performance, for example, an association between increased gamma activity and faster reaction times during motor tasks (Rieder et al., 2011).

Binocular rivalry (e.g., Doesburg et al., 2009) and masking studies (e.g., Fisch et al., 2009) have shown the association between conscious recognition of visual stimuli with gamma activity. In their electrocorticography study, Fisch et al. (2009) presented a briefly-flashed target image (16 ms), a blank screen of varying duration, and a mask. The duration of the blank screen determined whether the target image was sent either to a conscious or unconscious level. Participants categorized the target images into classes (faces, houses, objects). Gamma power after the onset of the target image was higher on successful recognition trials than on incorrect trials, supporting the positive relationship between gamma during encoding and conscious recognition of encoded stimuli. Further, this gamma power increase was immediate and sustained over higher-order visual areas after target image presentation, even when visual input was replaced by a masking stimulus. This suggests that the gamma increase reflects the interpretation process involved in stimulus recognition.

Thus, it is possible that expertise effect manifested in the gamma ERS in both dancers and non-dancers reflects higher-order cognitive operations in listening to their own music, selectively attending the stimulus, maintaining the representation of the music in WM, identifying the musical structure of the pieces, or consciously recognizing their own music. However, when listening to their own music, a lower gamma ERS was observed in the dancer group relative to the non-dancer group. This result may be explained by stimulus predictability of familiar (tango) music in dancers. It has been shown that gamma activity during selective attention to stimuli decreases with higher predictability of stimuli (Bauer et al., 2014). It is possible that dancers were planning/imagining dance movements while listening to their tango music. For dancers, the higher predictability of tango music may be advantageous for planning dance movements, explaining their relatively lower gamma activity to non-dancers.

Alternatively, increases in gamma and beta may have resulted from heightened emotional provocation from the familiarity of the music that, in the current design, participants had chosen as their favorite pieces. Both groups reported enjoying their own music more than the other music (Figure 3A). This is in line with the finding that familiarity with a musical piece enhanced the intensity of self-reported emotional responses to the piece (Ali and Peynircioglu, 2010). The relationship between higher frequency bands and emotion has been found in previous work. Gamma and beta activity was demonstrated to be a reliable indicator of liking music (Hadjidimitriou and Hadjileontiadis, 2012). This is in line with work reporting that high-arousing emotional stimuli (faces depicting angry expressions) or scenes (pleasant or unpleasant) elicit higher gamma activity compared to their less arousing counterparts (neutral faces or scenes) (Keil et al., 2001; Balconi and Lucchiari, 2008). In music listening, both gamma and beta were shown to be the best classifiers of different levels of emotions (Lin et al., 2010). In professional musicians, emotional responsiveness to music was found to be enhanced, characterized by an increase in beta and alpha activity (Mikutta et al., 2014). In our study, affective enjoyment had no relation with either beta or alpha activity. Rather, an affective relationship was observed only with gamma ERS (Figure 3B). The gamma ERS could have been due to a larger affective impact from their own music. It is possible that listening experience of a specific type of music strengthened the listeners' emotional responsiveness to the music.

Although our analysis and interpretation of the relationship between gamma and emotion is consistent with the previously established connection between higher frequency bands and affective responses, the beta-emotion relationship is less clear, as our multiple regression analysis did not find a correlation between beta ERS and enjoyment rating (Figure 3B). However, we still found significant beta increase in both groups while listening to their own music (Figure 2B, Table 1B). It is possible that the beta increase was brought about by characteristics intrinsic to their own music, such as tempo or rhythm, and not solely from an emotional connection. In a study examining the relationship between individual musical tempo preference to spectral frequency (Bauer et al., 2015), participants, who were enthusiasts of the rock genre of music, manually adjusted the tempo of rock music excerpts to a rhythm that was “just right,” yielding a mean value of preferred tempo for each person. EEG for different frequency bands was then analyzed in separate tasks: alpha while at rest with eyes closed, gamma while listening to 1 kHz sine tones presented at random interstimulus periods, and motor beta activity during a finger tapping task with a fixed rate. The personally preferred musical tempo was positively correlated with the individual peak of frequency only in the beta band during the motor finger tapping task, indicating that individual tempo preference is associated with beta activity, and not alpha or gamma.

The results of Bauer et al. (2015) suggest that the beta ERS in our study has association with tempo, in addition to or instead of emotional arousal. In our study, there was no main effect of Music (tango vs. non-tango) in the mixed ANOVA in the beta band, indicating that music characteristics specific to either genre alone, such as tempo, did not play a measurable role in the increase of beta activity. The combination of both findings, Bauer et al. and ours, indicates that *purely tempo itself in any music* was not the sole contributor to the increase of beta activity in our data. Rather, it is possible that the beta increase results from tempo *in the context of their preferred music*. All participants in Bauer et al.'s study were enthusiasts of rock, the genre of the musical stimuli used to determine their preferred tempo. Thus, despite the absence of physical movement, our participants' cognitive motor activity may have harmonized with the tempo of their preferred music. The harmonization may be stronger in dancers: dancers had higher beta ERS compared to non-dancers for their own music (Figure 2B). As they listen to music, dancers may more readily generate a mental template of tempo in order to successfully synchronize with the tempo of the music, a process dancers go through when they engage in a dance.

### Expertise Effect in the Alpha Band

Alpha activity appears in a relaxed state of mind (Jasper and Carmichael, 1935). In particular, alpha activity increases over the visual scalp area when eyes are closed (Pfurtscheller et al., 1996) as the thalamo-cortical gating system blocks the visual information flow to the occipital visual processing area (Pfurtscheller, 2003). In this study, EEGs were recorded with eyes closed. It is possible that the alpha activity in this study was largely driven by the deactivation of visual neurons. The analysis of Electrode in alpha ERS in this study identified three occipital electrodes (O1, Oz, O2) as most active. Thus, it is further possible that while listening to the music, participants were particularly relaxed to the point of falling asleep. Sleep studies document that alpha waves are prominent right before sleep onset (for a detailed review, see Klimesch, 1999). However, these explanations are unlikely in our study because all participants concurrently showed increased beta and gamma activity for their own music, which indicates a conscious state of mind and attention-driven awareness (Womelsdorf and Fries, 2007).

Rather, the increased alpha activity in the posterior region may indicate attentive cognitive operations in line with what is observed in experts such as elite athletes when they perform active motor or cognitive tasks (Del Percio et al., 2009; Duru and Assem, 2018), professional musicians while they listen to affectively-provoking segments of classical music (Mikutta et al., 2014), and experienced meditators during meditation (Buzsáki, 2006). All these studies reported increased alpha activity in the posterior region. Within this framework, when the dancers and non-dancers in our study listened to music, they engaged in cognitive processing of music as experienced listeners.

In experts, the increased posterior activity may spread into the surrounding regions. For example, with increased experience, alpha extends from the occipital region toward the parietal and central regions in meditators. The thalamo-cortical gating mechanism (Pfurtscheller, 2003) implies that the increased alpha activity in the posterior to middle scalp regions reflects an active disengagement from environmental inputs, e.g., visual, auditory, and somatosensory. This permits intense internal mental operations to occur in non-sensory areas (Buzsáki, 2006). The relationship between the active disengagement in sensory areas and engagement of non-sensory areas has been formalized as the alpha functional inhibition hypothesis (e.g., Jensen and Mazaheri, 2010; Klimesch, 2012). That is, the activity of alpha is an inhibitory mechanism of neural activity that occurs in task-irrelevant areas, and allows the allocation of resources to neural areas necessary for the task. In this respect, the alpha activity marking cortical deactivation is not the historically characterized “cortical idling,” but is instead regarded “functional” in that it actively supports mental operations.

We propose that the alpha functional inhibition hypothesis accounts for the expertise effects displayed by the dancers and non-dancers in our study, namely the initial alpha ERD and later alpha increase (see Figure 2A). Unlike gamma, the alpha increase did not emerge immediately after music onset. Instead, both groups showed a significant decrease in alpha activity for their own expertise-related music relative to other music in the first interval. This initial alpha ERD indicates that participants simultaneously exhibited an immediate release from inhibition (Klimesch, 2012) and an engagement of cortical activity upon hearing their own music. This engaged state of cortical activity may also have been reflected by the immediate gamma ERS shown by both groups for their own music. Simultaneous appearance of alpha ERD and gamma ERS in the first interval is one crucial piece of evidence underlying the alpha functional inhibition hypothesis, whereby alpha reflects the deactivation and gamma the activation of cortical areas during task engagement (Jensen and Mazaheri, 2010). After its initial ERD, alpha activity increased in later time intervals in both groups when they listened to their own music. Following the alpha functional inhibition hypothesis, the rise in alpha activity reflects the involvement of cognitive operations housed in anterior cortical regions. The cognitive expertise-related music processes in our dancers and non-dancers benefited from the inhibition of cortical activity in the task-irrelevant posterior region.

The functional role of alpha activity in sustaining cognitive operations is supported by a number of WM studies. In these studies, for example, an increase of number of visual stimuli (e.g., consonant letters or pictures of faces) to be remembered at a later time led to a parametric increase of parieto-occipital alpha activity during a retention period (e.g., Jensen et al., 2002; Tuladhar et al., 2007; Scheeringa et al., 2009). The once-increased alpha power rapidly diminished at the appearance of the probe (Jensen et al., 2002), indicating a close relationship between WM retention processes and alpha activity. Further, the parametric alpha power increase was related to a decrease in fMRI blood oxygen level dependent (BOLD) signals in the primary visual cortex (Scheeringa et al., 2009), supporting alpha activity's involvement in the deactivation of cortical areas. Jensen et al. (2002) hypothesize that the disengagement of parieto-occipital areas serves to suppress input from the visual stream, which disturbs the maintenance of working memory in anterior areas.

There was no specific task given in the current study, leaving us to postulate the content of cognitive operations in our dancers and non-dancers. Whatever they were, the processes were likely supported by mechanisms of attention or WM. A few possible processes associated with listening to their own music are suggested by the post-session interview (see Supplemental Material). A number of dancers responded that they imagined dancing or dancing in a *milonga*, a social gathering centered on Argentine tango dancing. Several non-dancers imagined instruments being played or musicians performing. This suggests that participants were involved in some cognitive operation related to how they experience their own music.

### Differences Between Dancers and Non-dancers in the Alpha Band

Differences between dancers and non-dancers emerged in the magnitude of effects in both the initial alpha decrease and later alpha increase when listening to their own music. A group-wise comparison of dancers and non-dancers (Figure 2A; Table 1A) revealed that in the first interval, the magnitude of ERD in dancers was larger than non-dancers. In all subsequent intervals, the magnitude of alpha ERS in dancers was larger than non-dancers. A music-wise comparison (Figure 2A; Table 1B) revealed dancers showed a robust music effect, wherein the alpha activity during listening to their own music was significantly different from listening to the other group's music. Although both groups showed a music effect with lower alpha activity for their own music compared to other group's music in the first interval, only dancers showed music effects during all subsequent intervals (Table 1B). Specifically, dancers showed higher alpha activity for their own music compared to other group's music, exhibiting a continuous music effect from the second to the fifth interval, lasting for 80s. In non-dancers, music effects with higher alpha activity for their own music emerged only in the third and fourth intervals, lasting for 40s.

Within the alpha functional inhibition hypothesis, the stronger initial alpha ERD in dancers may reflect a more effective release from alpha inhibition and thus suggests more rapid involvement with cortical activity. The subsequent alpha ERS that was sustained over later intervals in dancers suggests sustained cognitive engagements in the entire duration of listening to tango music. We postulate possible cognitive processes that might have occurred in dancers, which may have contributed to the dancer effect.

One of the possible processes is motor imagery (MI), wherein the dancers imagined tango dance movements either kinesthetically or visually. That is, feeling the movement through imagined sensations or self-visualizing the movement and postures, without actual performance (Hétu et al., 2013; Karin, 2016). Dance training involves memory retrieval of practiced movements with music and the application of imagined movements onto actions (Ermutlu et al., 2015). Controlled attention in accessing stored information is accompanied by alpha increase (Klimesch, 2012). Our dancers' increased alpha activity, therefore, may reflect retrieval processes of tango dance movements or forms.

In a meta-analysis of MI studies with fMRI and PET, Hétu et al. (2013) found that the supplementary motor area (SMA) in the frontal lobe is particularly involved in MI and in the planning of internally generated sequential movements. In the framework of the alpha functional inhibition hypothesis, it is possible that our dancers attentively recruited the frontal area to generate tango dance movements, by routing resources from the parieto-occipital region that was inhibited for visual sensory processing. Findings in an fMRI study, by Sacco et al. (2006) support this possibility. During an MI of walking, their participants showed both an increased activation in the premotor area (including SMA) and reduced activation in the posterior visual spatial area after five days of tango dance lessons.

An MI study with athletes with classical ballet training (dancers) and athletes with no dance training (non-dancers) revealed alpha and beta EEG increases in dancers (Ivaldi et al., 2017), similar to our study. Participants were instructed to make a kinesthetic MI to fit classical, rock, and waltz music. Dancers presented higher alpha activity for all three music types, and higher beta activity for classical. Their findings support our speculation that our dancers may have engaged in MI while listening to their own music. Unlike our results, however, in Ivaldi et al.'s study, the dancers' alpha and beta increase was found in the left hemisphere, suggesting hemisphere differences in kinesthetic MI between dancers and non-dancers.

Another process that might be relevant in our dancers is creative cognition. A strong positive association between creativity and alpha activity has been documented in a review of creativity and EEG (Stevens and Zabelina, 2019). Argentine Tango is an improvisational social dance. The dancers in the current study may have engaged in crafting their own dance movements or creating the social scene and environment of a milonga. Responses to the post-session interview “dancing in a milonga” from several dancers may support this idea. This is in line with a long-term memory encoding study by Meeuwissen et al. (2011), in which participants were encouraged to create sentences as a strategy to remember the order of words given in a list. Optimal memory performance was accompanied by strong alpha activity in the parieto-occipital region. The improvisational sentence-making processes share the basis of creative cognition with the improvisational creation of dance scenes.

Fink et al. (2009) put forward the idea of enhanced creativity in dancers as indexed by alpha activity. Professional dancers showed a high level of alpha activity in the posterior region when imagining an improvisational dance but no such alpha activity when imagining dancing a ballroom waltz with monotonous fixed-step patterns. The same dancers also showed a strong alpha activity in a divergent thinking task which measures creative cognitive processes. Fink et al. suggest that the dancers' increased alpha activity may be associated with frequent practices of creative expression. Thus, our dancers' alpha ERS may reflect enhanced creative ability, in the imagining of tango dance within its full context of a social setting rather than the access to rote-memorized movements.

It is possible that creative abilities benefit further from suppressing sensory input. It has been demonstrated that performance in divergent thinking tests improved when individuals closed their eyes (Ritter et al., 2018). It has also been reported that people skilled in divergent thinking were more adept at suppressing external stimuli that might hinder creative processes (Stevens and Zabelina, 2019). Together, these findings imply that creativity benefits from functionally inhibited cortical areas that process sensory information and thus accompanies alpha activity. The alpha ERS of dancers in our study suggests that not only did they have enhanced creative abilities, but were better able to suppress external stimuli than non-dancers.

Camarda et al. (2018) presents another aspect of the creativity-alpha relationship. They found increased alpha activity during divergent thinking tasks was sustained longer by people who performed better in these tasks, suggesting that sustained attention plays a key role in creative processes. The sustained alpha activity in our dancers, specifically the enhanced long lasting alpha ERS, thus suggests a dancers' enhanced ability to sustain attention that may originate from their dance-related experiences.

## Limitations of the Current Study and Future Directions

Higher resting-state alpha activity has been found in modern dancers compared to athletes and controls (Ermutlu et al., 2015). Dance-specific training, such as coordinating body movements with music and internalizing motor experiences by imagery and memory, was proposed to increase the resting-state alpha through innate mechanisms or changes in neural plasticity. Thus, dance training or long-term exposure to dance-related experiences appears to contribute to the general augmentation of attention. It would be of interest, in a future study, to measure the relationship of expert dancers' alpha activity with the level of task difficulty, for example, different levels of danceability in music. This would test whether a positive correlation exists between sustained attention and cognitive demand in dancers.

Our results show that dancers' sustained alpha activity in later intervals was higher than in non-dancers, not only when listening to tango music but also when listening to non-tango music (Figure 1A, Table 1A). Non-dancers did not show this pattern. We hypothesize that dancers may be expanding their cognitive ability of engaging in functional alpha inhibition from the processing of their tango music to the processing of music in general. That is, dance experiences may shape general cognitive skills, in this case, how to process music. It would be of interest to pursue this hypothesis as a further investigation of dance expertise effects.

Based on the apparent emotion-gamma relationship in both dancers and non-dancers, we further hypothesize that the observed gamma ERS can be explained by the combination of conscious recognition of familiar music and emotional arousal. However, the current design did not separate prior familiarity from liking, nor control the participant's prior exposure to a music genre, as we intended each group to be the expert of their own music per our primary research question. Future work should address these limitations by presenting music that is equally novel across groups, or by statistically controlling for familiarity.

## Conclusions

Expertise, characterized by extensive experience with listening to certain music, was reflected in an increase of gamma, beta, and alpha oscillatory activity. Increased EEG activity was observed when Argentine tango dancers listened to their favorite tango dance music, and when non-dancers listened to their favorite jazz or classical music. The gamma and beta ERS may reflect listeners' expertise in the processing of familiar music with attention and conscious recognition. Our multiple regression analysis of ERS with listener's enjoyment ratings found that emotional responsiveness to the music was reflected only in the gamma band. Beta increase may arise from both emotional arousal and tempo associated with their favorite music. An alpha ERD-ERS pattern in both dancers and non-dancers while listening to their favorite music fits the framework of a functional alpha inhibition model. Years of experience in listening to their favorite type of music may have honed the cerebral responses to achieve efficient cortical processes by means of increasing alpha activity. In addition to music-listening expertise, dancers exhibited a dance expertise effect, wherein a strong initial alpha ERD was followed by a consistent and sustained alpha ERS for 80s while listening to dance music. This pattern may suggest an alpha inhibition mechanism enhanced by the practice of creative cognition, including motor imagery, that results from a substantial experience in dance.

## Data Availability Statement

The raw data supporting the conclusions of this article will be made available by the authors, without undue reservation.

## Ethics Statement

The studies involving human participants were reviewed and approved by the Institutional Review Board of Saint Mary's College of California. The patients/participants provided their written informed consent to participate in this study.

## Author Contributions

HN and CdD designed the research. CdD collected the data. All authors analyzed the data and wrote the manuscript.

## Conflict of Interest

The authors declare that the research was conducted in the absence of any commercial or financial relationships that could be construed as a potential conflict of interest.

## Footnotes

^1^We included all electrodes in the analysis, including midline (z) electrodes. Though the z electrodes are placed over the interhemispheric fissure, which arguably has no activity, they capture the summated activity from neighboring cortical and subcortical areas as well. It is common for z electrodes to be significantly active. For example, Yuval-Greenberg et al. (2008), identified electrode CPz as having maximal gamma-band power in their time-frequency analysis.

## References

[B1] Adrian E. D. Matthews B. H. C. (1934). The Berger rhythm: potential changes from the occipital lobes in man. Brain A. J. Neurol. 57, 355–385. 10.1093/brain/57.4.355 20058345

[B2] Ali S. O. Peynircioglu Z. F. (2010). Intensity of emotions conveyed and elicited by familiar and unfamiliar music. Music Percept. 27, 177–182. 10.1525/mp.2010.27.3.177

[B3] Babiloni C. Marzano N. Infarinato F. Iacoboni M. Rizza G. Aschieri P.. (2010). “Neural efficiency” of experts' brain during judgment of actions: a high-resolution EEG study in elite and amateur karate athletes. Behav. Brain Res. 207, 466–475. 10.1016/j.bbr.2009.10.034 19891991

[B4] Bachorik J. P. Bangert M. Loui P. Larke K. Berger J. Rowe R.. (2009). Emotion in motion: investigating the time-course of emotional judgments of musical stimuli. Music Percept. 26, 355–364. 10.1525/mp.2009.26.4.355

[B5] Balconi M. Lucchiari C. (2008). Consciousness and arousal effects on emotional face processing as revealed by brain oscillations. A gamma band analysis. Int. J. Psychophysiol. 67, 41–46. 10.1016/j.ijpsycho.2007.10.002 17997495

[B6] Bauer A.-K. R. Kreutz G. Herrmann C. S. (2015). Individual musical tempo preference correlates with EEG beta rhythm. Psychophysiology 52, 600–604. 10.1111/psyp.12375 25353087

[B7] Bauer M. Stenner M.-P. Friston K. J. Dolan R. J. (2014). Attentional modulation of alpha/beta and gamma oscillations reflect functionally distinct processes. J. Neurosci. 34, 16117–16125. 10.1523/JNEUROSCI.3474-13.2014 25429152 PMC4244475

[B8] Berger H. (1929). Über das elektroenkephalogramm des menschen. Arch. Psychiatr. Nervenkr. 87, 527–570. 10.1007/BF01797193

[B9] Buzsáki G. (2006). Rhythms of the Brain. New York, NY: Oxford University Press. 10.1093/acprof:oso/9780195301069.001.0001

[B10] Camarda A. Salvia É. Vidal J. Weil B. Poirel N. Houd,é O.. (2018). Neural basis of functional fixedness during creative idea generation: an EEG study. Neuropsychologia 118, 4–12. 10.1016/j.neuropsychologia.2018.03.009 29530800

[B11] Carozzo S. Garbarino S. Serra S. Sannita W. G. (2010). Function-related gamma oscillations and conscious perception. J. Psychophysiol. 24, 102–106. 10.1027/0269-8803/a000019

[B12] Chase M. H. Harper R. M. (1971). Somatomotor and visceromotor correlates of operantly conditioned 12–14 c/sec sensorimotor cortical activity. Electroencephalogr Clin Neurophysiol 31, 85–92. 10.1016/0013-4694(71)90292-6 4105848

[B13] Cohen J. (1988). Statistical Power Analysis for the Behavioral Sciences. Hillsdale, N.J.: L. Erlbaum Associates.

[B14] Del Percio C. Babiloni C. Marzano N. Iacoboni M. Infarinato F. Vecchio F.. (2009). “Neural efficiency” of athletes' brain for upright standing: a high-resolution EEG study. Brain Res. Bull. 79, 193–200. 10.1016/j.brainresbull.2009.02.001 19429191

[B15] Delorme A. Makeig S. (2004). EEGLAB: an open source toolbox for analysis of single-trial EEG dynamics including independent component analysis. J. Neurosci. Methods 134, 9–21. 10.1016/j.jneumeth.2003.10.009 15102499

[B16] Doesburg S. M. Green J. J. McDonald J. J. Ward L. M. (2009). Rhythms of consciousness: binocular rivalry reveals large-scale oscillatory network dynamics mediating visual perception. PLoS ONE 4:e6142. 10.1371/journal.pone.0006142 19582165 PMC2702101

[B17] Duru A. D. Assem M. (2018). Investigating neural efficiency of elite karate athletes during a mental arithmetic task using EEG. Cogn. Neurodyn. 12, 95–102. 10.1007/s11571-017-9464-y 29435090 PMC5801285

[B18] Ermutlu N. Yücesir I. Eskikurt G. Temel T. Işoglu-Alkaç Ü. (2015). Brain electrical activities of dancers and fast ball sports athletes are different. Cogn. Neurodyn. 9, 257–263. 10.1007/s11571-014-9320-2 25834650 PMC4378580

[B19] Fink A. Graif B. Neubauer A. C. (2009). Brain correlates underlying creative thinking: EEG alpha activity in professional vs. novice dancers. Neuroimage 46, 854–862. 10.1016/j.neuroimage.2009.02.036 19269335

[B20] Fisch L. Privman E. Ramot M. Harel M. Nir Y. Kipervasser S.. (2009). Neural “ignition”: enhanced activation linked to perceptual awareness in human ventral stream visual cortex. Neuron 64, 562–574. 10.1016/j.neuron.2009.11.001 19945397 PMC2854160

[B21] Grabner R. H. Stern E. Neubauer A. C. (2003). When intelligence loses its impact: neural efficiency during reasoning in a familiar area. Int. J. Psychophysiol. 49, 89–98. 10.1016/S0167-8760(03)00095-3 12919712

[B22] Hadjidimitriou S. K. Hadjileontiadis L. J. (2012). Toward an EEG-based recognition of music liking using time-frequency analysis. IEEE Trans. Biomed. Eng. 59, 3498–3510. 10.1109/TBME.2012.2217495 23033323

[B23] Haegens S. Osipova D. Oostenveld R. Jensen O. (2010). Somatosensory working memory performance in humans depends on both engagement and disengagement of regions in a distributed network. Hum. Brain Mapp. 31, 26–35. 10.1002/hbm.20842 19569072 PMC6871021

[B24] Hétu S. Grégoire M. Saimpont A. Coll M.-P. Eugène F. Michon P.-E.. (2013). The neural network of motor imagery: an ALE meta-analysis. Neurosci. Biobehav. Rev. 37, 930–949. 10.1016/j.neubiorev.2013.03.017 23583615

[B25] Ivaldi M. Cugliari G. Fiorenti E. Rainoldi A. (2018). Delta and alpha rhythms are modulated by the physical movement knowledge in acrobatic gymnastics: an EEG study in visual context. Sport Sci. Health 14, 563–569. 10.1007/s11332-018-0461-2

[B26] Ivaldi M. Cugliari G. Peracchione S. Rainoldi A. (2017). Familiarity affects electrocortical power spectra during dance imagery, listening to different music genres: independent component analysis of alpha and beta rhythms. Sport. Sci. Health 13, 535–548. 10.1007/s11332-017-0379-0

[B27] Jasper H. H. Carmichael L. (1935). Electrical potentials from the intact human brain. Science 81, 51–53. 10.1126/science.81.2089.51 17801487

[B28] Jensen O. Gelfand J. Kounios J. Lisman J. E. (2002). Oscillations in the alpha band (9–12 Hz) increase with memory load during retention in a short-term memory task. Cereb. Cortex 12, 877–882. 10.1093/cercor/12.8.877 12122036

[B29] Jensen O. Mazaheri A. (2010). Shaping functional architecture by oscillatory alpha activity: gating by inhibition. Front. Hum. Neurosci. 4:186. 10.3389/fnhum.2010.00186 21119777 PMC2990626

[B30] Jung T.-P. Makeig S. Humphries C. Lee T.-W. McKeown M. J. Iragui V.. (2000). Removing electroencephalographic artifacts by blind source separation. Psychophysiology 37, 163–178. 10.1111/1469-8986.3720163 10731767

[B31] Karin J. (2016). Recontextualizing dance skills: overcoming impediments to motor learning and expressivity in ballet dancers. Front. Psychol. 7:431. 10.3389/fpsyg.2016.00431 27047437 PMC4805647

[B32] Keil A. Müller M. M. Gruber T. Wienbruch C. Stolarova M. Elbert T. (2001). Effects of emotional arousal in the cerebral hemispheres: a study of oscillatory brain activity and event-related potentials. Clin. Neurophysiol. 112, 2057–2068. 10.1016/S1388-2457(01)00654-X 11682344

[B33] Klimesch W. (1999). EEG alpha and theta oscillations reflect cognitive and memory performance: a review and analysis. Brain Res. Rev. 29, 169–195. 10.1016/S0165-0173(98)00056-3 10209231

[B34] Klimesch W. (2012). Alpha-band oscillations, attention, and controlled access to stored information. Trends Cogn. Sci. 16, 606–617. 10.1016/j.tics.2012.10.007 23141428 PMC3507158

[B35] Lin Y. Wang C. Jung T. Wu T. Jeng S. Duann J.. (2010). EEG-based emotion recognition in music listening. IEEE Trans. Biomed. Eng. 57, 1798–1806. 10.1109/TBME.2010.2048568 20442037

[B36] Luke S. G. (2017). Evaluating significance in linear mixed-effects models in R. Behav. Res. Methods 49, 1494–1502. 10.3758/s13428-016-0809-y 27620283

[B37] Meeuwissen E. B. Takashima A. Fernández G. Jensen O. (2011). Increase in posterior alpha activity during rehearsal predicts successful long-term memory formation of word sequences. Hum. Brain Mapp. 32, 2045–2053. 10.1002/hbm.21167 21162031 PMC6870165

[B38] Mikutta C. A. Maissen G. Altorfer A. Strik W. Koenig T. (2014). Professional musicians listen differently to music. Neuroscience 268, 102–111. 10.1016/j.neuroscience.2014.03.007 24637097

[B39] Müller M. M. Keil A. (2004). Neuronal synchronization and selective color processing in the human brain. J. Cogn. Neurosci. 16, 503–522. 10.1162/089892904322926827 15072684

[B40] Orgs G. Dombrowski J.-H. Heil M. Jansen-Osmann P. (2008). Expertise in dance modulates alpha/beta event-related desynchronization during action observation. Eur. J. Neurosci. 27, 3380–3384. 10.1111/j.1460-9568.2008.06271.x 18598273

[B41] Pavlova M. Birbaumer N. Sokolov A. (2006). Attentional modulation of cortical neuromagnetic gamma response to biological movement. Cereb. Cortex 16, 321–327. 10.1093/cercor/bhi108 15901655

[B42] Pfurtscheller G. (2003). Induced oscillations in the alpha band: functional meaning. Epilepsia 44, 2–8. 10.1111/j.0013-9580.2003.12001.x 14641556

[B43] Pfurtscheller G. Stancák A. Neuper C. (1996). Event-related synchronization (ERS) in the alpha band —an electrophysiological correlate of cortical idling: a review. Int. J. Psychophysiol. 24, 39–46. 10.1016/S0167-8760(96)00066-9 8978434

[B44] Rieder M. K. Rahm B. Williams J. D. Kaiser J. (2011). Human gamma-band activity and behavior. Int. J. Psychophysiol. 79, 39–48. 10.1016/j.ijpsycho.2010.08.010 20828587

[B45] Ritter S. M. Abbing J. van Schie H. T. (2018). Eye-closure enhances creative performance on divergent and convergent creativity tasks. Front. Psychol. 9:1315. 10.3389/fpsyg.2018.01315 30108537 PMC6079281

[B46] Sacco K. Cauda F. Cerliani L. Mate D. Duca S. Geminiani G. C. (2006). Motor imagery of walking following training in locomotor attention. The effect of ‘the tango lesson.' Neuroimage 32, 1441–1449. 10.1016/j.neuroimage.2006.05.018 16861008

[B47] Scheeringa R. Petersson K. M. Oostenveld R. Norris D. G. Hagoort P. Bastiaansen M. C. M. (2009). Trial-by-trial coupling between EEG and BOLD identifies networks related to alpha and theta EEG power increases during working memory maintenance. Neuroimage 44, 1224–1238. 10.1016/j.neuroimage.2008.08.041 18840533

[B48] Stevens C. E. Zabelina D. L. (2019). Creativity comes in waves: an EEG-focused exploration of the creative brain. Curr. Opin. Behav. Sci. 27, 154–162. 10.1016/j.cobeha.2019.02.003

[B49] Tuladhar A. M. Huurne N. ter Schoffelen J.-M. Maris E. Oostenveld R. Jensen O. (2007). Parieto-occipital sources account for the increase in alpha activity with working memory load. Hum. Brain Mapp. 28, 785–792. 10.1002/hbm.20306 17266103 PMC6871495

[B50] Womelsdorf T. Fries P. (2007). The role of neuronal synchronization in selective attention. Curr. Opin. Neurobiol. 17, 154–160. 10.1016/j.conb.2007.02.002 17306527

[B51] Yuval-Greenberg S. Tomer O. Keren A. S. Nelken I. Deouell L. Y. (2008). Transient induced gamma-band response in EEG as a manifestation of miniature saccades. Neuron 58, 429–441. 10.1016/j.neuron.2008.03.027 18466752

