# Supplementary material for: Experience Affects EEG Event-Related Synchronization in Dancers and Non-dancers While Listening to Preferred Music
Source: Front Psychol. 2021 Apr 12;12:611355. doi: 10.3389/fpsyg.2021.611355 (PMC8071982; doi:10.3389/fpsyg.2021.611355)
Supplement: Supplementary file 1 [file Table_1.DOCX]

**Supplemental Material**

Participants were interviewed about their experience after the EEG session. Researchers read questions directly from a written script to each participant, and noted the substance of the participant’s response. The table below provides the questions and types of responses, with the number of each kind of response indicated in the parenthesis.

| DANCER | NONDANCER |
| --- | --- |
| 1. What were you thinking while you were listening to (tango/classical/jazz…preferred) music? | |
| **Referencing tango music (own music)**   - Themselves dancing, envisioning dancing, dancing in a milonga (6) - Moving (1) - Analyzing the music, rhythm, instruments, or actively listening to the music (4) - Visualizing colors, people, objects (2) - Actively trying not to think (1) | **Referencing nontango music (own music)**   - Actively listening or enjoying the music (3) - Imagining instruments, the band, or a desire to sing (3) - Movement or dancing (1) - Visualizing images (1) - Stories related to music (1) - Relaxing or not actively thinking (2) |
| 1. Follow-up: Was there a song that brought back a particular memory or made you remember something? | |
| **Referencing tango (own music)**   - Buenoes Aires and milongas (1) - Doing a performance and choreography (1) - Their country (1)   **Referencing nontango**   - Swingy jazz brought back a memory (1) - Assessed the music, e.g. predictable, enjoyable, pretty, or noting they learned about it in school (2) - Imagined going to a piano bar (1)   **Not referencing a genre**   - Visualized faces, colors, or unspecified images (2) - Images of orchestra with dancers (1) - Interactions with people, unspecified incident (1) - The first time they heard the song (1) | **Referencing tango**   - A trip to Argentina (1)   **Referencing nontango (own music)**   - Imagining people listening to music, playing instruments, or couples dancing (3) - Watching a movie/show associated with the song, a scene in a movie (2) - Liked a piece best because they play an instrument featured in that piece; followed the piece because they know the structure (2) - When they got the CD of the song (1) - Reading a book while listening to the piece (1) - A person they know playing the piece on violin (1) - Fast paced song reminded them of high school (1) - An unspecified association (1)   **Not referencing a genre**   - Recalled the lyrics and artist (1) - Visualized people dancing (1) |
| DANCER | NONDANCER |
| 1. Did you pay attention to the music, or listen to the music like it was background noise? How did you pay attention to the music? Did you listen to specific instruments/melodies/rhythm? | |
| **Referencing tango (own music)**   - Listened to the emotional flow of the music (1) - Paid attention, unspecified imagining (1)   **Referencing nontango**   - Listened actively, e.g. to specific instruments, beat, melody (4) - Paid attention sometimes, thoughts drifted (1)   **Not referencing a genre**   - Listened actively, e.g. to specific instruments (violins, piano), melody, rhythm (5) - Paid attention (2) - Critiqued or compared music (2) - Imagined instruments, dancing (2) - Imagined people at a wedding, unspecified imagery (2) - Trying to relax (1) - Paid attention sometimes, thoughts drifted to landscape images and orchestra, (1) | **Referencing tango**   - Listened actively, e.g. to the piano, bandoneon, structure, a general sense of the music (4) - Couldn’t pay attention or tuned it out (2)   **Referencing nontango (own music)**   - Listened actively, e.g. to instruments (horns, the bass, the piano, cello,), melody, or identifying the piece (6) - Hard for it to be background noise (1)   **Not referencing a genre**   - Listened actively to intervals and rhythmic changes, instruments (violins, trumpet, piano) (4) - Paid attention then drifted, or music became background noise (2) - Paid attention especially to those that reminded them of childhood (1) - Felt like moving their body, e.g. tapping hands (1) |
| 1. Overall, which song did you like best? Was there a song you did not like? | |
| **Referencing tango (own music)**   - Specific tango song as favorite, with a reason e.g. the speed (9)   **Referencing nontango**   - Specific jazz piece liked, felt like walking in Europe or surprised them (2) - Specific classical piece liked (1) - Jazz less appealing, but still liked (1) - Jazz disliked (1)   **Not referencing a genre**   - Specific song disliked for various reasons such as boring showtune-like, too slow (6) - No song they didn’t like, just liked less, or wasn’t great (4) | **Referencing tango**   - Specific tango song, or all tango disliked, some with reasons such as chaotic, discordant, or sounded strange (8) - Specific tango song liked (2)   **Referencing nontango (own music)**   - Specific classical piece liked (5) - Specific jazz piece liked (3) - Specific jazz piece disliked (1)   **Not referencing a genre**   - Specific song disliked, boring (1) - No song they didn’t like, liked them all (1) |
| DANCER | NONDANCER |
| 1. Did you find any music distracting? | |
| **Referencing nontango (own music)**   - Xylophone playing was distracting (1) - Difficult to focus, but not distracting (1) - A specific classical piece (1)   **Not referencing a genre**   - Nothing distracting (5) - Nothing distracting, but some boring or reminded of a period piece/movie (2) - Everything distracting (1) | **Referencing tango**   - Tango pieces, some with reasons such as sounding random or sharp, out of tune, couldn’t relax (5)   **Referencing nontango (own music)**   - Singing the lyrics to their songs (1) - Preferred music, because more at ease (1)   **Not referencing a genre**   - Nothing distracting (4) - Specific song (2) - Everything distracting (1) - Faster pieces made them want to move (1) |
| 1. Any comments on particular music? | |
| **Referencing tango (own music)**   - Specific piece very familiar or in ingrained (2) - Liked specific piece, but not as a piece to dance to (1)   **Referencing nontango**   - Enjoyed classical piece, some wanted to hear more (2) - Felt like dancing to jazz (1) - Familiar with classical piece (1) - Interesting to hear 30s-40s music (1) - Detached from jazz (1)   **Not referencing a genre**   - Enjoyed the mix of music (1) - Volumes a little off (1) - Liked some songs because of bass or wanting to dance (2) | **Referencing tango**   - Gets you moving, tried to dance or envisioned dancing routines (2) - Liked a specific tango, excited because it’s not usual type of music, like dramatic music (2) - Tango ok (1)   **Referencing nontango (own music)**   - Thinking of words to one of the broadway pieces (1) - Swing pieces evocative, familiar with style (1) - Listen to wide variety of jazz (1)   **Not referencing a genre**   - Like happy music (1) - Accordions can be used well or poorly (1) - Couldn’t distinguish between music pieces I didn’t choose(1) - Some songs were nice or ok (2) |
| DANCER | NONDANCER |
| 1. Did you feel uncomfortable at any time (i.e. wanting to move your body, tired, etc.)? | |
| **Referencing nontango (own music)**   - Started to move body (1)   **Not referencing a genre**   - Swallowing or trying to control swallowing (3) - Bodily functions: stomach growling, bladder full (3) - Falling asleep, getting tired (2) - Twitchy or itchy (2) - Wanted to dance (1) - Couldn’t lean head back, neck stress (1) | **Referencing tango**   - Unspecified “reaction” to tango (1) - Didn’t feel like dancing (1)   **Referencing nontango (own music)**   - Wanted to dance or move to jazz (1)   **Not referencing a genre**   - Annoyed couldn’t lean head back, neck tense (4) - Lower back uncomfortable, tired of sitting (2) - Bodily functions: hungry, bladder full (2) - Falling asleep, getting tired (2) - Cap felt tight (1) - Eyes moving a lot (1) - Wanted to dance (1) |
| 1. Did you feel sleepy/tired/bored towards the end? | |
| **Referencing tango (own music)**   - Sleepy, but last tango woke me up (1)   **Referencing nontango**   - A little sleepy during last jazz song (1)   **Not referencing a genre**   - Yes (2) - Relaxed and sleepy, not bored (1) - Bored and hungry (1) - Excitement from anticipating the next song (1) - No, settling down for a long session (1) | **Referencing nontango (own music)**   - Jazz music mellows me out, wanted to fall asleep (1) - Excited to hear my songs (1)   **Not referencing a genre**   - Sleepy or tired (5) - Relaxed, not sleepy (1) - Looking forward to listening to music (1) - Wide awake, last song brought energy up (1) - Boring to listen to music they didn’t like (1) - Antsy due to neck tension (1) |
